# The role of procalcitonin and presepsin in the septic febrile neutropenia in acute leukemia patients

**DOI:** 10.1371/journal.pone.0253842

**Published:** 2021-07-29

**Authors:** Rania Moustafa, Taissir Albouni, Ghassan Aziz

**Affiliations:** 1 Department of Laboratory Medicine, Faculty of Medicine, Damascus University, Damascus, Syria; 2 Department of Internal Medicine (Hematology), Faculty of Medicine, Damascus University, Damascus, Syria; University of Kentucky, UNITED STATES

## Abstract

**Background:**

The source of bacterial infection in neutropenic acute leukemia patients is detected in about 20–30% of cases. Bacterial cultures may require a long incubation period and risk false-positive and false- negative results. Therefore, biomarkers distinguishing septic febrile neutropenia from other etiologies in acute leukemia patients play the important role in patient assessment and treatment planning. This study aims to determine the role of procalcitonin (PCT) and presepsin (PSPN) in infectious complication in comparison to C-reactive protein (CRP) on the first and third day at the onset of febrile neutropenia in patients with acute leukemia.

**Methods:**

Between June 2018 and February 2019, 60 acute leukemia patients with febrile neutropenia receiving chemotherapy. The 41 acute myeloid leukemia patients and 19 acute lymphoblastic leukemia patients were recruited in this study. Their ages ranged from 14 to 65 years. PCT and PSPN were measured and were compared to CRP at the onset of febrile neutropenia and after 48 hours. 20 patients had a fever of unknown origin (FUO) and 40 patients had a bacterial infection.

**Findings:**

Our results showed that the values of these markers were higher in patients with infection than patients without. The area under the curve (AUC) of PCT were 0.931 and 0.813 on day one and three respectively, which was the best in determination of infection. The cut-off values of PCT were 1.27 and 1.23 ng/mL and the cut off values of PSPN were 1.75 and 2.9 μg/L in the successive days, their clinical sensitivities were high. PCT and PSPN were capable of distinguishing the cause of febrile neutropenia from the onset of infection and predicting its complications (p<0.05). The PSPN level couldn’t differentiate gram-positive or gram-negative bacterial infection. Significant differences were found between the mean values of the PSPN during the successive days in all patients and patients with bacteremia. This study illustrated a weak positive correlation between PCT and Sequential Organ Failure Assessment (SOFA) score, the negligible correlation between CRP and SOFA score and no significant correlation between PSPN and SOFA score.

**Interpretation:**

PCT is an accurate biomarker in identifying infection in acute leukemia patients, its concentration is associated with the severity of bacterial sepsis. PSPN is superior to PCT for follow-up of patients.

## Introduction

Febrile neutropenia is a serious complication of chemotherapy in acute leukemia patients. Infection is one of the most common leading causes of morbidity and mortality in patients with neutropenic acute leukemia [1]. Fever is not a specific symptom of infection in such patients; it may be due to the tumor itself or chemical drugs. Hence, it’s important to detect the cause of fever in order to determine the appropriate therapy to manage these complications [2]. The source of infection in patients with neutropenic fever has been identified in about 20–30% of cases [3]. Bacterial cultures need a relatively long time to give results accompanied by positive or negative falsehoods of the culture results. So biomarkers could play an important role in determining the cause of fever in neutropenic acute leukaemia patients. There are many biomarkers to be studied to assess their usefulness in determining infection [4].

The C-reactive protein (CRP) is the acute phase protein; however it has low specificity for infections. It is the most commonly used inflammatory markers in the clinical practice due to its low cost in a laboratory analysis in the clinical practice. It rises in inflammatory and infectious diseases; it works as a comparative marker with other biomarkers [5].

Procalcitonin (PCT) is a peptide precursor of the calcitonin hormone, consisting of 116 amino acids, first mentioned in the medical literature as a marker of sepsis in 1993, produced from the C cell of thyroid [6]. PCT is synthesized in the liver, muscle, adipose, lung, kidney and intestine cells during the inflammatory and infectious injury, either directly as a result of contact with toxins of pathogens or indirectly in response to inflammatory cytokines whose secretion is induced due to burns, tumours and infections [7]. Therefore, PCT is not specific to infections. It rises to a high level in the bacterial infection and elevates during 2–4 hours of injury, reaching a peak within 24 hours, then it returns to normal levels in case of response to treatment within 2–3 days [8]. Many studies have shown that PCT is a vital marker possesses high sensitivity and specificity for the diagnosis of infections injury and follow up of disease progression [9–11].

CD14 (Cluster of differentiation) has an important role in the process of antibacterial phagocytosis. It is a glycoprotein found mainly on the surface of phagocytes (monocytes, neutrophils), B lymphocytes, epithelial cells, parenchymal cells in the liver and intestinal [12]. It is a member of the PRR (Pattern recognition receptor) receptor family in cell membranes and acts as a receptor for polysaccharide complex and other compounds in Gram-negative and positive bacteria. Once activated, the signal is transmitted to the cytoplasm and after successive activations the cell induces the secretion of inflammatory cytokines. It has two forms, the first form mCD14 associated with the membrane of the cell and the second form sCD14 which is soluble in plasma. Presepsin (PSPN) is a part of sCD14 (sCD14-subtype) that consists of 64 amino acids [13]. It’s a new biomarker is used in diagnosing infection and follow up of patients. Its plasma concentration is an indicator of active natural immunity as an early defensive line against invasive pathogens, as it is produced by the liver as part of acute-phase proteins [12]. PSPN rises early within two hours of inflammation. It has a high sensitivity and specificity to bacterial infections.

Several studies included heterogeneous groups of hematological malignancy patients with conflicting findings about the role of PCT and PSPN in identifying sepsis [14–17]. Therefore, it is necessary to have the laboratory methods that help in determining the cause of fever in acute leukemia patients, consequently the appropriate treatment, especially in the Middle East region where the mortality rates of leukemia prevalence is increasing, due to the infections caused by multi drug-resistant bacteria. Therefore, we selected a sample of neutropenic acute leukaemia patients with a high risk of infection to study the role of PCT and PSPN in early identification of infection comparative to a conventional one that used in clinical practice.

## Materials and methods

### Study design

Patients and parental consent were obtained if the patients were under the age of 18 years old. This study was done according to Declaration of Helsinki (Research approval was obtained from the Committee of Research Ethics in Health Colleges at Damascus University).

This prospective study included 60 neutropenia acute leukemia undergoing chemotherapy who were admitted to the department of hematology and infectious diseases at Al-Mowasat University hospital between July 2018 and February 2019. We evaluated the role of PCT and PSPN in detecting septic febrile neutropenia. Blood samples were drawn at the onset of the febrile neutropenia and on the third day (48 hours after the onset of febrile neutropenia).

Inclusion criteria are as follows: patients with acute leukemia, at the age ≥14 years, with febrile neutropenia, which fever was defined as a single oral temperature of 38.3°C or over or a temperature of 38.0°C or over for 1h or more and neutropenia was defined as a neutrophil count less than 0.5×10^9^/L or a neutrophil count of <1.0×10^9^/L in patients whose neutrophil counts were expected to decline to <0.5×10^9^/L [18]. Exclusion criteria: patients who received antibiotics within 24 hours before to calibration of laboratory markers and patients with hepatic or renal damage when the values of these markers are higher in patients with these injuries [19].

Based on Sepsis-3 criteria, bacterial sepsis was defined as confirmed bacterial infection with ≥ 2 or more points from the baseline in SOFA (Sequential Organ Failure Assessment) score, depended on six different score, one each for the hepatic, respiratory, coagulation, cardiovascular, renal and neurological systems. Septic shock was defined as sepsis induced hypotension <65 mm Hg with hyperlactatemia >18 mg/dL, despite fluid replacement [20, 21].

**Fig 1:**
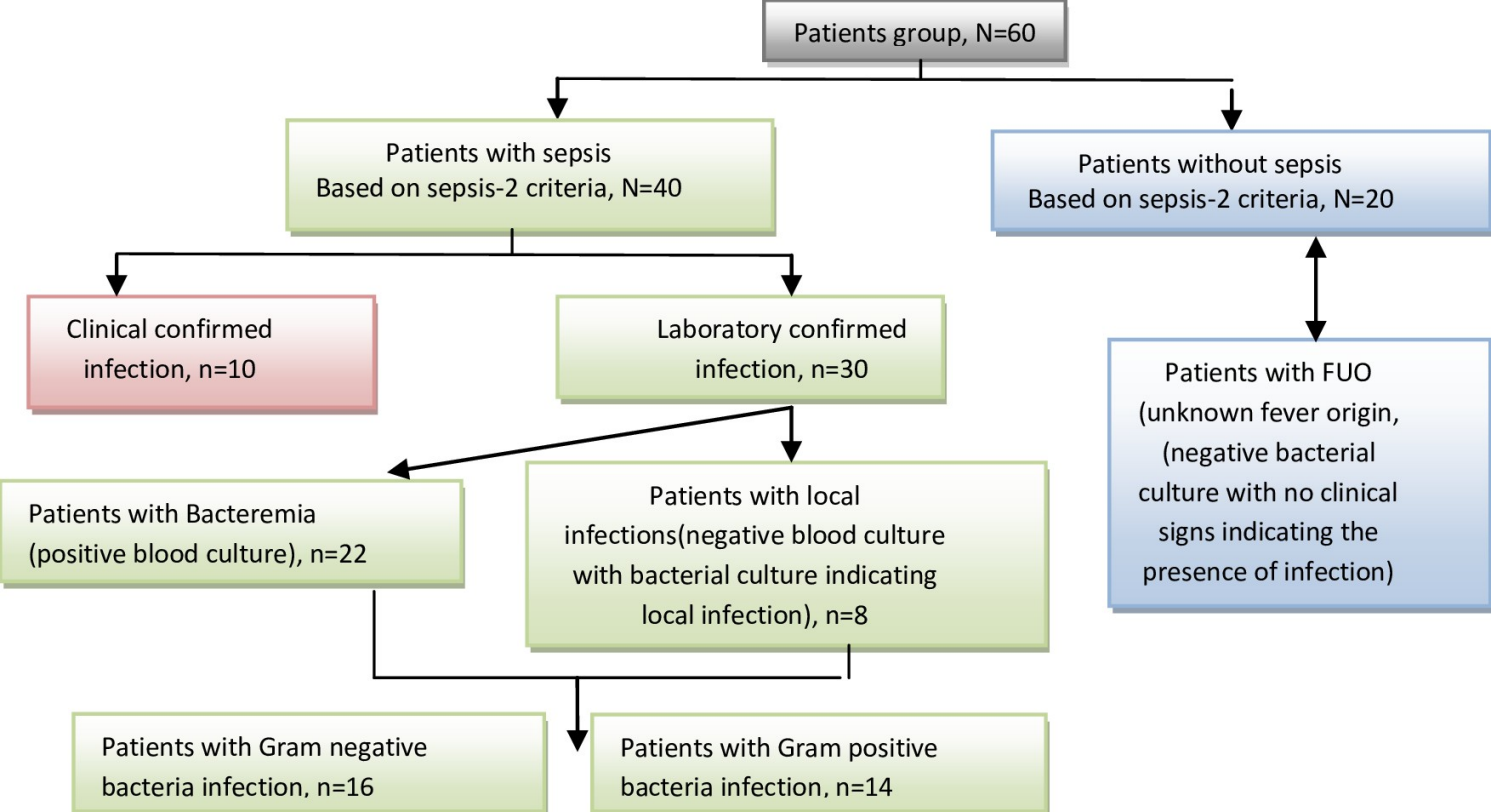
Distribution of patients’ groups were according to bacterial infection.

The medical data was collected in the special questionnaire, including the patient’s age, type of leukemia, vital signs, oxygen saturation, clinical examination, and chest x-ray from the patient’s records in the hospital. Patients were divided into groups according to bacterial infection (Fig 1).

### Laboratory methods

Bacterial cultures were performed before antibiotic therapy to determine pathogenic bacteria in clinical samples (sputum, urine, pus and blood). Blood samples were collected from all patients in the EDTA tube for absolute neutrophil count and platelet. A dry tube to obtain serum kept them at -20° until all samples were collected. All the analyses samples were collected on 1^st^ day and 3^rd^ day to determine the concentration of biomarkers. CRP level was measured by the immune turbidimetric method by a device Response ®920, using kit (CRP FS, Diasys Diagnostic Systems, Germany). PCT level was measured by direct immunofluorescence assay by a device I-chroma using a kit (Boditech Med Inc, Korea). Assay rang: 0.1–100 ng/mL. PSPN level was measured by ELISA (double antibody sandwich enzyme linked immunosorbent assay) using a kit (SunRed, Shanghai, China). The optical density of the sample and the standard was measured by a plate reader (450 nm), using the device Multiskan EX. We determined the optical density of each standard against its predetermined concentration in a logarithmic diagram then we drew the curve with benchmarks that were calculated in serological samples. Assay rang:0.03–9 μg/l.

We determined the value of PSPN positive if it was more than 0.314 μg/L, 0.5 ng/mL for PCT and was 5 mg/L for CRP according to manufacturer’s instructions.

### Statistical analysis

The sample size was calculated depending on the following formula: *n = (p)x(1-p)x(z/e)*^*2*^. Where (n: sample size, p: the probability of matching the sample to the statistical community, z: the critical standard degree, e: the permissible error). The sample size was 60 patients at the level of significance 7.5% and confidence interval 92.5%.

The results were analyzed using the descriptive-analytical method in SPSS (version22) to detect the relationship between study variables. Continuous variables were determined as means with standard error, as medians with a rang (minimum and maximum), Student t-test, one way ANOVA test and Pearson test were used to compare averages of the biomarkers. Statistical tests were carried out at a significance level of 0.05. Descriptive curves (Receiver Operation Characteristics (ROC) curve analysis) were analyzed to assess the accuracy of biomarkers in differentiating between the patients with and without infection. The sensitivity, specificity, positive predictive, negative predictive and diagnostic values of PSPN, PCT and CRP were determined.

## Results

### Patients

We investigated the diagnostic and predictive role of PCT and PSPN in comparison to CRP in 60 patients with neutropenia acute leukemia at the onset of febrile neutropenia. As shown in Table 1, we determined bacterial infection in 40 patients (18 patients with local infection and 22 patients with bacteremia) diagnosed by clinical examination suggesting infection and growth in bacterial cultures (blood, urine, sputum, pus).

**Table 1:** Characteristics of the study patients and sites of infection.

**General**			
Male (n, %)	32(53.3%)		
Age (range, median)	14–65 years, 15.98±34.92		
Type of leukemia (n, %) /AML	41(68.3%)		
Febrile neutropenia			
Neutrophil (mean, range)	134.5 cell/μl (10–460 cell/μl)		
Clinical syndrome*	**Sample**	**Pathogens**	**Number**
**Bacteremia**	Blood	Gram-negative bacteria	
		*Escherichia coli*	6
		*Klebsiella pneumonia*	3
		*Pseudomonas aeruginosa*	1
		Gram-positive bacteria	
		*Staphylococcus aureus*	4
		*Staphylococcus epidermis*	4
		*Streptococcus* spp	4
**Laboratory confirmed local infection**	Urine	*Escherichia coli*	3
	Sputum	*Escherichia coli*	2
		*Klebsiella pneumonia*	1
	Pus	*Staphylococcus epidermis*	2
**Clinical confirmed local infection**		Pneumonia^1^	7
		Gastrointestinal infections^2^	3

*Confirmed bacterial infection was known as a clinical infection and identification of relevant bacteria by bacterial culture.

1. Presence of radiographic signed up the bacterial pneumonia with negative sputum culture.

2. Clostridium difficile toxin test was positive in three patients who were suffering from diarrhea.

### The role of PSPN and PCT in diagnosing infection

Firstly, the patients were divided into three groups to study the role of PSPN and PCT in distinguishing the cause of febrile neutropenia. The values of the biomarkers of the three groups illustrated in (S1 Fig in S1 File). The respective mean levels were as follows: 2 and 3.2 ng /mL for PCT, 2.4 and 3.9 μg/L for PSPN for fever of unknown origin group. 7.4 and 6. 8ng/mL for PCT, 3.3 and 3.7 μg/L for PSPN for local infection group. 16.7 and 14 ng/mL for PCT, 4 and 5.7 μg/L for PSPN for bacteremia group on the first and third day. Table 2 shows a detailed overview of the data.

**Table 2:** Values of biomarkers in groups of patients according to the cause of febrile neutropenia.

Days	Biomarker	Patient’s group	N	Mean	Std. Deviation	95% Confidence Interval for Mean		Min	Max	ANOVA test (F =)
						Lower Bound	Upper Bound			
**The first day**	**PSPN (μg/L)**	**FUO**	20	2.4	1.4	1.8	3.1	0.9	5.4	8.97
		**Local infection**	18	3.3	1.2	2.7	3.9	1.8	5.5	
		**Bacteremia**	22	4	1.2	3.5	4.5	2.6	6.8	
	**PCT (ng/mL)**	**FUO**	20	2	2.2	1	3	0.17	9.8	36.6
		**Local infection**	18	7.4	7.5	3.6	11.1	0.1	34.6	
		**Bacteremia**	22	16.7	9.3	12.6	20.8	4.56	45.3	
	**CRP (mg/L)**	**FUO**	20	72.1	43.8	51.5	92.6	8.9	164.7	37.17
		**Local infection**	18	77.6	37	59.2	96	21.9	154	
		**Bacteremia**	22	110.7	20	101.8	119.6	67.8	140	
**The third day**	**PSPN (μg/L)**	**FUO**	20	3.9	1.9	3	4.8	0.4	6.8	8.11
		**Local infection**	18	3.7	1.9	2.8	4.7	0.5	6.1	
		**Bacteremia**	22	5.7	1.2	5.2	6.3	3.2	6.8	
	**PCT (ng/mL)**	**FUO**	20	3.2	4.7	0.9	5.4	0.1	15.2	28.12
		**Local infection**	18	6.8	5.4	4.2	9.5	0.1	20	
		**Bacteremia**	22	14	9.9	9.6	18.4	2.1	39	
	**CRP (mg/L)**	**FUO**	20	72.4	57.3	45.5	99.2	1.2	152.5	31.51
		**Local infection**	18	102.2	57.2	73.7	130.6	14	237.9	
		**Bacteremia**	22	99.9	54.4	75.7	124	13.7	280.6	

CRP, C-reactive protein; PCT, Procalcitonin; PSPN, Presepsin; N, Number of cases; FUO, Fever of unknown origin; min, minimum; max, maximum.

By using the ANOVA test, there was a statistically significant difference between the PSPN and PCT values according to different patient groups (FUO, local infection, and bacteremia), (p<0.05), compared to CRP which was unable to distinguish the cause of the febrile neutropenia in the three groups on the first day (p = 0.205).

Next, we divided our patients into two groups (patients without confirmed infection and patients with confirmed infection). By using the t-test, there were statistically significant differences for mean values of PCT, PSPN and CRP between the two groups, and this difference was in favor of the patient group with infection. On the first day, the mean of PCT level differed for patients with and without infection (12.5ng/mL versus 2ng/mL respectively, where t = -6.541; p = 0.000). PSPN differed for patients with and without infection (3.7 μg/L versus 2.4 μg/L where t = - 0.369; p = 0.001). CRP differed for patients with and without infection (95.8 mg/L versus72.6 mg/L where t = -2.348; p = 0.022). On the third day, mean values of PCT differed for patients with and without infection (10.8 ng/mL versus 3.2 ng/mL respectively, t = -3.582; p = 0.001). Mean values of PSPN differed also (4.8 μg/L versus 3.9 μg/L, t = -1.818; p = 0.074). Mean values of CRP differed between two groups (100.9 mg/L versus72.4 mg/L respectively, t = -1.869; p = 0.067>0.05). This study aimed to identify infection in the early period of injury, so we focused on sensitivity rather than specificity.

The sensitivity and specificity of a series of possible cutting points for PCT and PSPN were calculated and these results were shown in the ROC curve in the (S2 Fig in S1 File), confidence interval was 95%. The analysis showed the area under the curve and cut-off values identified on two-time points as shown in Table 3. We calculated the sensitivity, specificity, positive predictive and negative predictive values of cut-off values for the PSPN, PCT and CRP that were useful for distinguishing septic febrile neutropenia in acute leukemia patients.

**Table 3:** Sensitivity, specificity, Negative Predictive Value (NPV), Positive Predictive Values (PPV) of cut- off values calculated over 2 days.

Days	Biomarker	Cut-off	AUC	Specificity	Sensitivity	NPV	PPV
**The first day**	**PSPN**	g/Lμ 1.75	0.766	%55	100%	%100	%81.6
	**PCT**	1.27 ng/mL	0.931	%50	%97.5	90.9%	79.6%
	**CRP**	83.3 mg/L	0.685	%75	%72.5	%57.7	%85.3
**The third day**	**PSPN**	2.9 μg/L	0.641	%35	%90	%63.6	%73.5
	**PCT**	ng/mL1.23	0.813	%45	%90	%69.2	%76.6
	**CRP**	mg/L36.3	0.631	%30	%90	%66.7	%75

PSPN: Presepsin, PCT: Procalcitonin, CRP: C-reactive protein, AUC: Area Under the Curve, NPV: Negative predictive value, PPV: Positive predictive value.

As shown in Table 3: PCT, PSPN, and CRP could be used as a criterion for distinguishing between a group of patients with infection and without infection, but PCT was the best from other in diagnostic infections (AUC, the closer this value is to one, it indicates the accuracy of the scale used). The mean values of the PSPN and CRP did not differ between Gram-positive and Gram negative bacterial infections (t-test, p>0.05). However, the mean values of the PCT differed statistically between Gram-positive and Gram negative bacterial infections on the third day in patients with infection and bacteremia group (t-test, p<0.05).

Returning to the Table 2 and using the one way ANOVA test to assess the role of biomarkers in the monitoring of patients during the infection. Significant differences were found between mean values of PSPN and PCT during the successive days in all patients (F = 6.236, 1.485, respectively p<0.05), while these significant differences were only found between the mean values of the PSPN during the successive days in the bacteremia group (F = 4.795, p = 0.012). We detected the correlations between the mean values of each pair of the biomarkers in patients of this study (S1 Text in S1 File).

### PSPN, PCT, CRP levels and SOFA score

Twelve patients (2 patients had a local infection and 10 patients had bacteremia) attained criteria of sepsis -3 (confirmed bacterial infection and changed in SOFA score ≥ 2 points) and no one developed septic shock at the onset of neutropenia. The mean values of PCT, PSPN, and CRP were reported on the first day of the patients’ group with and without bacterial sepsis. Mean PCT was found to be higher in patients with sepsis (n = 12, 18.64±11.9 ng/mL) in comparison to patients with non-sepsis (n = 48, 6.58± 6.84 ng/mL respectively). Mean PSPN was 4.03±1.06 μg/L in patients with sepsis and 3.08±1.44 μg/L in patients without sepsis. Also CRP differed for patients with and without sepsis (109.29 ± 32.21 mg/L versus 82.54±38.13 mg/L). By using Pearson correlation, there was no significant correlation between the PSPN values and SOFA score (r = 0.218, p>0.05). The correlation between CRP and SOFA score was negligible (r = 0.255, p = 0.05).While the results showed a low positive correlation between PCT and SOFA score (r = 0.457, p = 0.001), indicating that the values of PCT increased and the patient’s condition deteriorated clinically.

## Discussion

In this study, we found that patient with bacterial infection had a higher serum concentration of PCT, PSPN and CRP as well as in patient who had unknown cause fever. The PCT compared to other biomarkers played an important role as a vital marker for the diagnosis of infection according to ROC curve (AUC of PCT was 0.931, 0.813 on the first and third day). We have chosen the cut-off values with high sensitivity for identifying infection as 1.27 ng/mL for PCT and 1.75 μg/L for PSPN on the first day and 1.23 ng/mL for PCT, 2.9 μg/L for PSPN on the third day. Previous studies appeared that PCT was the best compared to PSPN [9, 22–25], while another study reported that PSPN was better than PCT [26]. We divided patients into three groups based on the reason of febrile neutropenia (unknown fever origin, local infection and bacteremia). The level of PSPN and PCT was the highest of bacteremia group in the two days and there were statistical differences between groups. PCT and PSPN were qualitative markers capable of distinguishing the cause of febrile neutropenia from the onset of injury and predicting its complications as other studies [22]. However, The low PCT and PSPN concentration of some patients with FUO do not mean stopping treatment with antibiotics. The low PCT and PSPN values are not sufficient to recommend doing so biomarkers were helpful tools in identifying infection. However, before the biomarkers’ roles are confirmed in larger studies, antibiotics treatment was only discontinued after clinical improvement. From the results of this study, the PCT and PSPN values were low in several patients despite having local infection and bacteremia. Although the average values of CRP on the first day were higher for the bacteremia group than patients with localized infection, it is a non-specific marker to distinguish the cause of febrile neutropenia and to predict fever complication at the onset of the injury within the first 24 hours. In this study, we calculated the average values that did not express on the response of each patient to infection. Our results could be related to the fact that samples were withdrawn for bacterial culture and biomarker titration on the first day before the patients’ treatments with antibiotic. After the treatment began, the average difference in values between patients was as a result of the patient’s response to antibiotic treatment directly against the bacteria causing infection. The measurement of PCT in the early stage of infection will not only help in assessing the severity of infection, but will also help in distinguishing the bacterium pattern and providing a basis for treatment. The current study showed that patients with Gram-positive bacterial infections had PCT levels higher than PCT levels with Gram-negative bacterial infections, with a statistically significant difference in the level of PCT between the two groups on the third day, the reason may be that patients with Gram-negative bacterial infections responded more quickly to treatment. This corresponds to the findings of other studies [9, 27]. While the values of PSPN and CRP did not differ statistically, it was necessary to keep in mind that these results did not reflect the condition of each patient. When we calculated the levels of biomarkers and by using t-test, PSPN was better than PCT for monitoring patients during infection and antibiotic treatment in the consecutive days at the onset of neutropenia. These series of assays were important tools in predicting fever complications and following up the treatment response [28, 29]. Sepsis criteria changed in the last years from Sepsis-2 (A systemic inflammatory response syndrome caused by an infection) to Sepsis-3 (A life-threatening organ dysfunction due to an unorganized host response to infection). Our results showed that PSPN levels were significantly higher in the group with sepsis than those without (4.03±1.06 vs3.08±1.44 μg/L), but the unexpected finding was no significant correlation between PSPN and SOFA score (r = 0.218, p>0.05) comparison to another study demonstrated significant correlation between PSPN and severity of fungemia [30]. This suggests that initial PSPN values are not predictive of organ dysfunction worsened in patients with bacterial sepsis. Also mean CRP found to be higher in patients with sepsis in comparison to patients with non-sepsis, and a negligible correlation was found between CRP level and SOFA score (r = 0.255, p = 0.05). PCT concentrations were higher in patients with sepsis than patients without sepsis (18.64±11.9 vs 6.58± 6.84 ng/mL, respectively). PCT and the degree of severity of sepsis demonstrated a weak positive correlation (r = 0.45, p = 0.01), accordingly, PCT helped predicting the progression of the clinical course of neutropenic fever to origin failure at the onset of neutropenia as one study [31]. There was a statistically significant correlation between the three markers on the first day (p<0.05). The correlation between the values of biomarkers was useful in increasing the sensitivity and quality of markers and might help in assessing the infectious injury. The difference of correlation between the markers was may be due to the different vital characteristics. It distinguished each marker especially the plasma half-life, marker values after stimulation, the different peak time and the different immune response to gene therapy for each patient; it played an important role [32]. However, there is no perfect marker yet [33].

The study was the single center, and we were not able to obtain biomarkers values to study the values of biomarkers before and after the febrile neutropenia. Also, the small sample size was the other limitations to be declared. We are fully aware of the limitation in the present study.

## Conclusions

Based on the results of this study, the PCT and PSPN were superior to CRP which used in the clinical practice to diagnose infection. There were some considerable complications, especially in immunosuppressed patients who were at high risk of infection. Our results showed that PCT was highly sensitive and specific in distinguishing the bacterial cause of septic fever from other causes. It helped predicting the complication of febrile neutropenia. PSPN was better when used as a follow-up marker. Therefore, combining both PCT and PSPN is better than conducting biomarker alone to manage infection in acute leukemia patients.

## Supporting information

S1 File (PDF)

S1 Data (XLSX)
