# Supplementary material for: The role of procalcitonin and presepsin in the septic febrile neutropenia in acute leukemia patients
Source: PLoS One. 2021 Jul 29;16(7):e0253842. doi: 10.1371/journal.pone.0253842 (PMC8321513; doi:10.1371/journal.pone.0253842)
Supplement: S1 File — (PDF) [file pone.0253842.s001.pdf]

PlOS ONE Supporting information

Article title: The role of procalcitonin and presepsin in the septic febrile neutropenia in acute leukemia patients.

Authors: Rania Moustafa, Taissir Albounii, and Ghassan Aziz.

The following information is available for this article:

S1 Fig: Distribution of the PCT,PSPN and CRP values according to the FUO, local infection and bacteremia group on the first and third day.

S2 Fig: Roc curve for PCT,PSPN and CRP values on the first and third day.

S1 Text: The correlation between of PSPN, PCT and CRP.

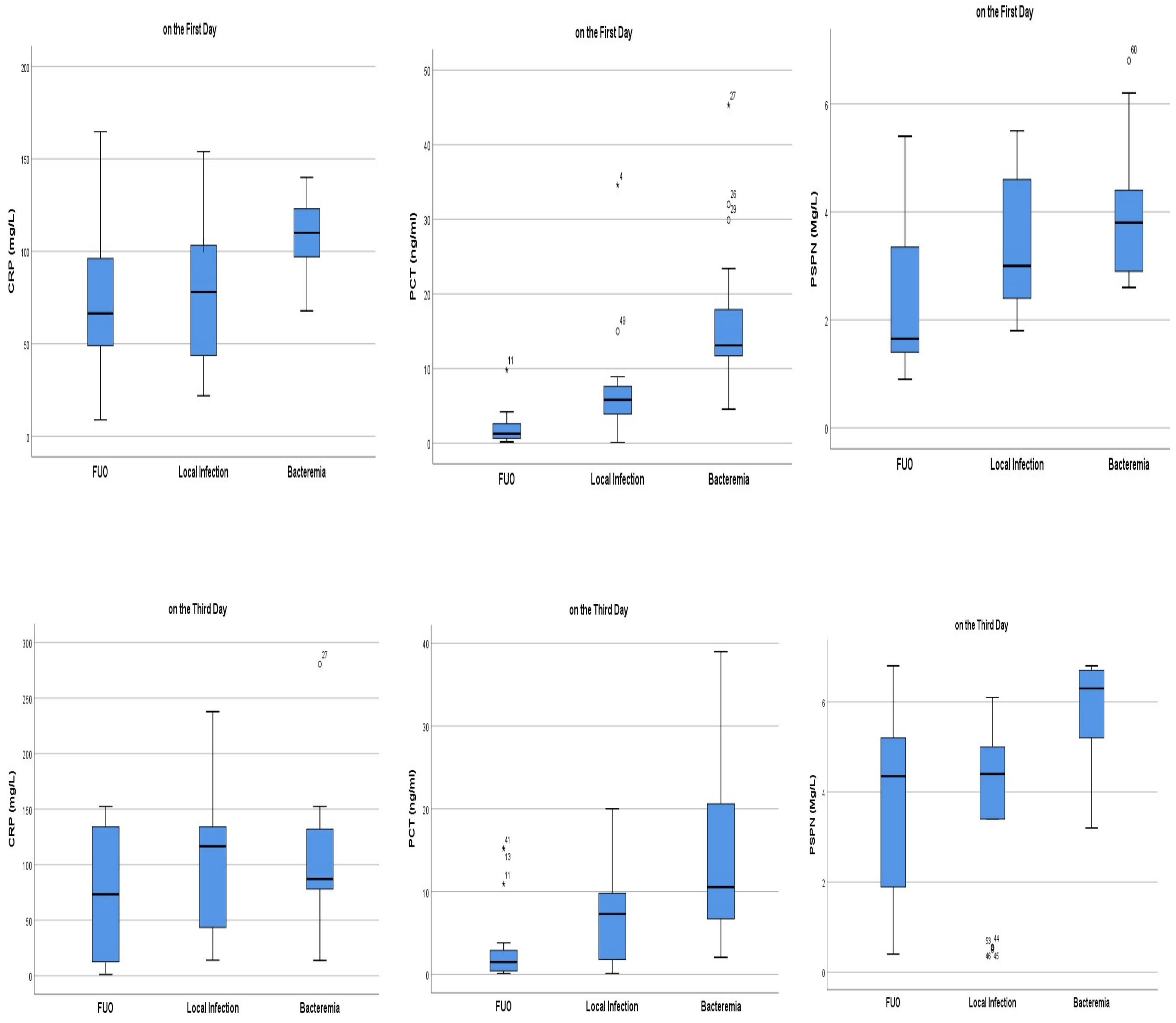

S1 Figure: Distribution of the PCT,PSPN and CRP values according to the FUO, local infection and bacteremia group on the first and third day.

These figures illustrated the three biomarkers values in the three patients groups that mentioned in the Table2. Mean of values for the three biomarkers were higher in infected groups than FUO group on the first and third day.

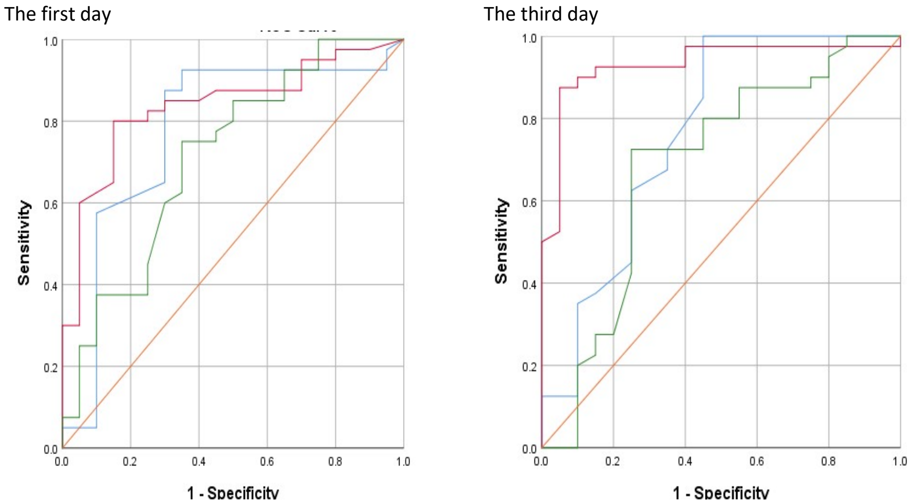

S2 Figure: Roc curve for CRP,PSPN and PCT values on the first and third day

PSPN, PCT, CRP and Reference. Area Under the Curve (AUC) of Presepsin (PSPN µg/L), Procalcitonin (PCT ng/mL) and C-reactive protein (CRP mg/L).

From the figures, AUC of PCT was higher than AUC of PSPN and CRP. It was the best to diagnosis infection.The sensitivity and specificity of a series of possible cutting points for PCT, CRP and PSPN were calculated, and the analysis showed the area under the curve during two days, confidence interval is 95%.

S1 Text: The correlation between of PSPN, PCT and CRP

Pearson correlation analysis was used to study if there are a relationship between the PSPN, PCT, and CRP values for all and bacteremia patients every day.

On the first day, negligible significant correlation was between the biomarkers (PCT& PSPN, PSPN&CRP) for all patients (r=0.262 and 0.282 respectively) and low positive significant

correlation between PCT& CRP(r=0.435), p.value < 0.05.There was the moderate correlation between CRP&PCT with bacteremia group (r=0.529) on the first day, p.value < 0.05.

On the third day, there was no correlation between each pair of biomarkers.
